# The Volvo 740 Turbo Estate

**Published:** 1988-02

**Authors:** Paul Goddard


### Critical Extravaganzas

**The Volvo 740 Turbo Estate**

"Warp factor nine" "Check" "We have blast off"

The dashboard lights flicked on in an amazing array. The onboard computer flashed messages about the fuel reserves and predicted time of arrival . . . and the stereo burst into beautiful sound. We truly were in orbit.

This was no ordinary car. In fact my children were sure that it was a starship.

Lex Brooklands had very kindly lent me their top of the range estate 740. With fuel injection and turbo it promised to be a very interesting weekend.

I picked up the car at 4.00 pm and drove back to the BRI. Parking the car in the RTC car park I was highly impressed by the manoeuvrability considering its large size . . . and the porters and early leaving staff were impressed by the customised body shell, Ferrari wheels and built in spotlights judging by their admiring looks and comments.

Friday evening we were off to a gig at the Anchor Inn, Bleadon. As usual we were playing with the other members of the Doctor Jazz Quartet in aid of the Bristol MRI Scanner Fund. One of our perennial problems is the transportation of equipment . . . synthesiser, amplification, boom stands etc. Tonight was different. The equipment fitted into the car with ample room to spare. We were even able to take full drum kit and the drummer.

More admiring comments. (That's some set of wheels, won the pools, like your new car etc, etc.)

Saturday: off to Croydon (140 miles) to pick up the children and play at another charity fundraising event. Off like a rocket down the M4. Nought to 60 in 7.7 seconds was entirely believable as the acceleration crushed us back against our seats. The turbocharged 2.3 litre engine delivered 182 bhp with amazing fuel economy for such a large car . . . around 32 mpg.

The maximum speed is not given by Volvo but one customer had been clocked for speeding at 138 mph!

Sunday: Many more admiring glances later and its the acid test . . . would my mother like the car? In the past she has turned up her nose at a variety of new cars and the only car in which she professed contetment when sitting in the back seat was a large Bentley.

Yes, it passed even this test. Not only was it smooooooth and comfortable but she felt completely safe as well. Nor was she immune from its good looks . . . she wanted me to order two!

Monday: The car is covered in frost but starts first time. I have to take it back, unfortunately, since I no longer wish to part with it.

I found the car very easy to drive despite having not previously driven a Volvo. The seats were fully adjustable including lumbar support and the controls well positioned. Extra fittings available through Lex Brooklands included anti-lock brakes, air conditioning, electric sunroof, bodykit, cruise control, trip computer and those amazing Ferrari wheels.

The price of the standard Volvo 740 Turbo estate is around £18,000 including car tax and VAT. But as for me . . . I'd take the optional extras. After all, what's another 3,000 or so for truly space age driving.

All in all this car is suitable for a doctor in many ways. Volvo have an admirable record for reliability and safety. The estate car is extremely spacious and with this model they have thrown off their staid image . . . it is a very presentable, no, let's be honest, enviable car.

"Beam me up, Scotty!"

Next Issue . . . I test drive an Audi, then it's back to Volvo for their new Sports Car. Watch this space!

Paul Goddard

